# Ciprofloxacin-Resistant *Salmonella enterica* Serovar Kentucky in Canada

**DOI:** 10.3201/eid1906.121351

**Published:** 2013-06

**Authors:** Michael R. Mulvey, David A. Boyd, Rita Finley, Ken Fakharuddin, Stacie Langner, Vanessa Allen, Lei Ang, Sadjia Bekal, Sameh El Bailey, David Haldane, Linda Hoang, Greg Horsman, Marie Louis, Lourens Robberts, John Wylie

**Affiliations:** Public Health Agency of Canada, Winnipeg, Manitoba, Canada (M. R. Mulvey, D. A. Boyd, K. Fakharuddin, S. Langner);; Public Health Agency of Canada, Guelph, Ontario, Canada (R. Finley);; Ontario Agency for Health Protection and Promotion, Toronto, Ontario, Canada (V. Allen);; Queen Elizabeth Hospital, Charlottetown, Prince Edward Island, Canada (L. Ang);; Institut National de Santé Publique du Quebec, Montreal, Quebec, Canada (S. Bekal);; Saint John Regional Hospital, Saint John, New Brunswick, Canada (S. El Bailey);; Provincial Public Health Laboratory Network of Nova Scotia, Halifax, Nova Scotia, Canada (D. Haldane);; British Columbia Centre for Disease Control, Vancouver, British Columbia, Canada (L. Hoang);; Saskatchewan Disease Control Laboratory, Regina, Saskatchewan, Canada (G. Horsman);; Alberta Provincial Laboratory for Public Health, Calgary, Alberta, Canada (M. Louis);; Newfoundland Public Health Laboratory, St. John’s, Newfoundland, Canada (L. Robberts);; Cadham Provincial Laboratory, Winnipeg, Manitoba (J. Wylie)

**Keywords:** ciprofloxacin resistance, multidrug resistance, antimicrobial resistance, Salmonella enterica serovar Kentucky, bacteria, Salmonella genomic island, Canada

## Abstract

We report emergence of ciprofloxacin-resistant *Salmonella enterica* serovar Kentucky in Canada during 2003–2009. All isolates had similar macrorestriction patterns and were multilocus sequence type ST198, which has been observed in Europe and Africa. Ciprofloxacin-resistant *S. enterica* serovar Kentucky represents 66% of all ciprofloxacin-resistant nontyphoidal *Salmonella* sp. isolates observed in Canada since 2003.

Infections with *Salmonella* spp. are a major health concern for humans and animals on a global scale. Although most cases of salmonellosis result in uncomplicated diarrhea, elderly and immunocompromised persons can be at risk for more severe invasive infections, which can be life-threatening and may require antimicrobial drug therapy (*1*). The drugs of choice for treating these invasive infections are fluoroquinolones (for adults) or cephalosporins.

One of the main drivers of antimicrobial drug resistance in *Salmonella* spp. is use of antimicrobial drugs in food-producing animals. For example, high rates of cephalosporin resistance in *Salmonella enterica* serovar Heidelberg isolated from poultry, retail chicken meat, and humans were observed in Quebec, Canada in 2003. After a voluntary withdrawal of cephalosporins was instituted by the Quebec broiler industry in 2005, rates of ceftiofur resistance dramatically decreased in animals and humans (*2*).

As with cephalosporin resistance, ciprofloxacin resistance in *Salmonella* spp. is a growing concern. Recently, *S. enterica* serovar Kentucky isolates have been described in Europe and Africa that were ciprofloxacin resistant (*3*). In addition, these isolates were resistant to multiple classes of antimicrobial drugs, which further complicates treatment options for invasive disease. No *S. enterica* serovar Kentucky isolates submitted to the National Antimicrobial Resistance Monitoring System in the United States were ciprofloxacin resistant (*3*). The purpose of this study was to describe the epidemiology and characterize isolates of ciprofloxacin-resistant *S.* *enterica* serovar Kentucky identified in Canada.

## The Study

The Canadian Integrated Program for Antimicrobial Resistance Surveillance (CIPARS), established in 2003, monitors antimicrobial drug use and resistance in selected species of enteric bacteria from humans, animals, and animal-derived food sources across Canada (www.phac-aspc.gc.ca/ cipars-picra/surv-eng.php). Human *Salmonella* isolates were submitted by all provincial public health laboratories in Canada to the National Microbiology Laboratory for further characterization. Antimicrobial drug susceptibility testing was performed by using broth microdilution (Sensititer Automated Microbiology System; Trek Diagnostic Systems Ltd., Westlake, OH, USA) and breakpoints established by the Clinical Laboratory Standards Institute (*4*).

**Figure 1:**
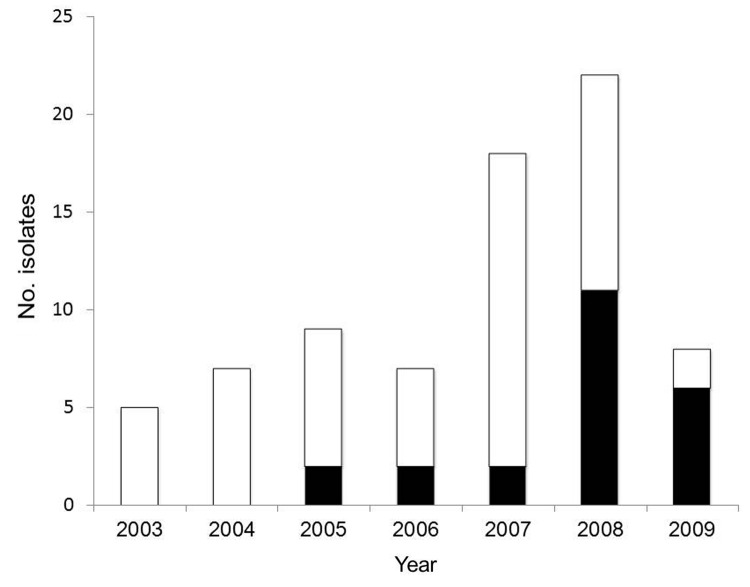
*Salmonella enterica* serovar Kentucky isolates identified in Canada, 2003–2009. Black bars indicate ciprofloxacin-resistant isolates, and white bars indicate non–ciprofloxacin-resistant isolates.

A total of 76 *S. enterica* serovar Kentucky isolates were submitted to the CIPARS program during 2003–2009, and 23 (30%) isolates showed ciprofloxacin resistance (MIC ≥4 mg/L) during the study (Figure 1). Thirty-five (46%) isolates were susceptible to all antimicrobial drugs tested. Ciprofloxacin-resistant isolates were identified from human case-patients in British Columbia (n = 2), Alberta (n = 2), Saskatchewan (n = 1), Ontario (n = 12), Quebec (n = 5), and Prince Edward Island (n = 1). Age information was available for 54 of 76 case-patients infected with *S. enterica* serovar Kentucky during the study period.

Of these isolates, 11 (14.5%) were resistant to ciprofloxacin. Ciprofloxacin resistance was observed among case-patients 18–69 years of age, and 5 of 11 were 18–29 years of age. Case-patients 18–29 years of age were 8 times more likely to have a ciprofloxacin-resistant strain than case-patients 50–69 years of age (odds ratio [OR] 8.3, 95% CI 1.034–67.198, p = 0.046). Of 21 ciprofloxacin-resistant isolates from case-patients who reported site of isolation, 20 were identified from feces and 1 from urine. There were no differences in site of isolation between ciprofloxacin-resistant and ciprofloxacin-susceptible *S. enterica* serovar Kentucky isolates. Although the total number of isolates associated with human infections was rare, of the 21,426 nontyphoidal *Salmonella* spp. submitted for susceptibility testing as part of the human component of the CIPARS program since 2003, *S*. enterica serovar Kentucky had a significantly higher rate of ciprofloxacin resistance than all other nontyphoidal *Salmonella* isolates and comprised 66% (23/35; p<0.0001) of all ciprofloxacin-resistant isolates identified during that period.

In Canada, ciprofloxacin-resistant *S. enterica* serovar Kentucky was first identified in 2005, when 22% (2/9) of isolates submitted for drug susceptibility testing were resistant to this drug. A significant increase (OR 10.5, 95% CI 1.115–9.913, p = 0.04) in the number of isolates resistant to ciprofloxacin was observed in 2009 compared with results in 2005. The largest number occurred during 2008–2009, when ciprofloxacin-resistant isolates comprised 57% (17/30) of all *S. enterica* serovar Kentucky isolates identified (Figure 1). The number of cases reported in Canada is comparable with that reported in Denmark over a similar period (*3*).

**Figure 2:**
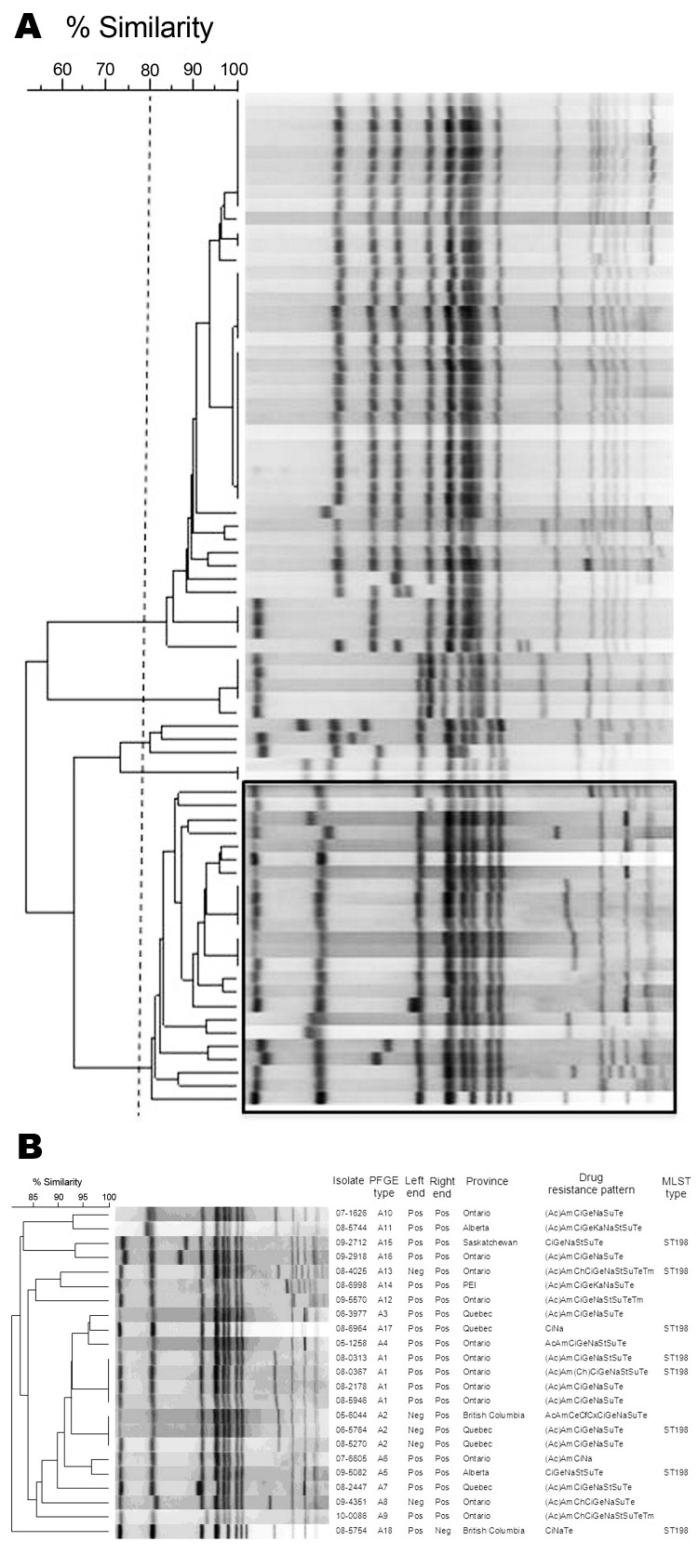
Dendrograms of macrorestriction fragments of all (A) and ciprofloxacin-resistant (B) *Salmonella* *enterica* serovar Kentucky isolates identified in Canada, 2003–2009. The dotted vertical line in panel A indicates a cutoff value of 80% similarity, and the box indicates ciprofloxacin-resistant isolates. Left end and Right end in panel B indicate PCR results for presence (Pos) or absence (Neg) of left and right junctions of *Salmonella* genomic island 1. PFGE, pulsed-field gel electrophoresis; MLST, multilocus sequence typing; Ac, amoxicillin clavulanate; Am, ampicillin; Ch, chloramphenicol; Ci, ciprofloxacin; Ge, gentamicin; Na, nalidixic acid; St, streptomycin; Su, sulfisoxazole; Te, tetracycline; ST, sequence type; Tm, trimethoprim; PEI, Prince Edward Island. Letters in parentheses indicate drugs that had intermediate MICs.

We typed all isolates by using pulsed-field gel electrophoresis as described and restriction enzyme *Xba*I (*5*). A dendrogram depicting the results was generated with BioNumerics version 3.5 (Applied Maths, Sint-Martens-Latem, Belgium) and is shown in Figure 2. All ciprofloxacin-resistant isolates clustered with a percentage similarity >80%; only 1 ciprofloxacin-susceptible isolate was found in this cluster (Figure 2, panel A).

Multilocus sequence typing (MLST) was performed on a subset of 8 isolates on the basis of differences in pulsed-field gel electrophoresis patterns and variations in antimicrobial drug resistance. Data were submitted to the MLST database website (http://mlst.ucc.ie/mlst/dbs/Senterica) to determine MLST types (*6*). All isolates tested were sequence type (ST) 198 (Figure 2, panel B). This sequence type and similar antimicrobial drug resistance patterns have been recently reported in France, England and Wales, Denmark, Belgium, and Africa (*3**,**7**,**8*).

Many ST198 multidrug-resistant isolates observed in Europe and Africa contained *Salmonella* genomic island 1 (SGI1) variants, particularly, SGI1-K, SGI1-Q, and SGI1-P. To determine whether ciprofloxacin-resistant isolates from Canada harbored similar SGI1 variants, we used PCR to detect the chromosomal left and right junctions of SGI1 as described (*9**,**10*). The right junction was found in 22 of 23 isolates, and the left junction was found in 18 of 23 isolates (Figure 2, panel B). Further studies are needed to identify specific SGI variants in the isolates.

Analysis of *S. enterica* serovar Kentucky isolates obtained during 2003–2009 from animal and retail meat samples as part of CIPARS did not identify any ciprofloxacin-resistant isolates (www.phac-aspc.gc.ca/cipars-picra/index-eng.php). This finding suggests that human infections in Canada were not acquired from domestically produced food. Many ciprofloxacin-resistant *S. enterica* serovar Kentucky human infections identified in Europe have been linked to travel to countries in Africa (*3*). Of 23 case-patients in Canada, we obtained travel history for 11. Travel history was defined as previous travel out of Canada within the past 7 days. Four case-patients had traveled to Morocco (1 also had traveled to Spain and Portugal), 3 had traveled to Egypt, 1 had traveled to Libya, and 3 had traveled to Africa (no country reported).

## Conclusions

Resistance to ciprofloxacin in *Salmonella* spp. is a growing concern because it limits the ability to treat invasive disease. In this study, we described the characteristics of ciprofloxacin-resistant *S. enterica* serovar Kentucky isolates in Canada. Similar drug-resistance patterns and genetic backgrounds of *S. enterica* serovar Kentucky have been observed in Europe and linked to travel to countries in Africa (*3*). That most isolates had multidrug resistance phenotypes is of particular concern. Further studies are required to determine risk factors for acquisition of these infections in Canada.

## References

[1] American Academy of Pediatrics. *Salmonella* infections. In: Pickering LK, editor. Red book: 2009 report of the committee on infectious diseases, 28th ed. Elk Grove Village (IL): American Academy of Pediatrics; 2009. p. 584.

[2] Dutil L, Irwin R, Finley R, Ng LK, Avery B, Boerlin P, Ceftiofur resistance in *Salmonella enterica* serovar Heidelberg from chicken meat and humans, Canada. Emerg Infect Dis. 2010;16:48–54. 10.3201/eid1601.090729 20031042 PMC2874360

[3] Le Hello S, Hendriksen RS, Doublet B, Fisher I, Nielsen EM, Whichard JM, International spread of an epidemic population of *Salmonella enterica* serotype Kentucky ST198 resistant to ciprofloxacin. J Infect Dis. 2011;204:675–84. 10.1093/infdis/jir409 21813512

[4] Clinical and Laboratory Standards Institute. Performance standards for antimicrobial susceptibility testing: eighteenth informational supplement. CLSI document M100–S18. Wayne (PA): The Institute; 2008.

[5] Ribot EM, Fair MA, Gautom R, Cameron DN, Hunter SB, Swaminathan B, Standardization of pulsed-field gel electrophoresis protocols for the subtyping of *Escherichia coli* O157:H7, *Salmonella*, and *Shigella* for PulseNet. Foodborne Pathog Dis. 2006;3:59–67. 10.1089/fpd.2006.3.59 16602980

[6] Harbottle H, White DG, Mcdermott PF, Walker RD, Zhao S. Comparison of multilocus sequence typing, pulsed-field gel electrophoresis, and antimicrobial susceptibility typing for characterization of *Salmonella enterica* serotype Newport isolates. J Clin Microbiol. 2006;44:2449–57. 10.1128/JCM.00019-06 16825363 PMC1489510

[7] Collard JM, Place S, Denis O, Rodriguez-Villalobos H, Vrints M, Weill F-X, Travel-acquired salmonellosis due to *Salmonella* Kentucky resistant to ciprofloxacin, ceftriaxone and co-trimoxazole and associated with treatment failure. J Antimicrob Chemother. 2007;60:190–2. 10.1093/jac/dkm114 17449886

[8] Weill F-X, Bertrand S, Guesnier F, Baucheron S, Cloeckaert A, Grimont PA. Ciprofloxacin-resistant *Salmonella* Kentucky in travelers. Emerg Infect Dis. 2006;12:1611–2. 10.3201/eid1210.060589 17176589 PMC3290958

[9] Boyd D, Peters GA, Cloeckaert A, Boumedine KS, Chaslus-Dancla E, Imberechts H, Complete nucleotide sequence of a 43-kilobase genomic island associated with the multidrug resistance region of *Salmonella enterica* serovar Typhimurium DT104 and its identification in phage type DT120 and serovar Agona. J Bacteriol. 2001;183:5725–32. 10.1128/JB.183.19.5725-5732.2001 11544236 PMC95465

[10] Doublet B, Praud K, Bertrand S, Collard J-M, Weill F-X, Cloeckaert A. Novel insertion sequence- and transposon-mediated genetic rearrangements in genomic island SGI1 of *Salmonella enterica* serovar Kentucky. Antimicrob Agents Chemother. 2008;52:3745–54. 10.1128/AAC.00525-08 18676889 PMC2565918

